# Assessment of Number of Cusps and Occlusal Groove Pattern in the Primary Mandibular Molars: An Observational Study

**DOI:** 10.31729/jnma.8935

**Published:** 2025-04-30

**Authors:** Megha Pradhan, Preeti Singh, Anu Ranjan, Ujjwal Joshi, Prasamsa Chhetri

**Affiliations:** 1 Department of Pedodontics ond Preventive Dentistry, Kathmandu Medical College ond Teaching Hospital, Sinamangal, Kathmandu, Nepal; 2 Department of Oral Pathology, Kathmandu Medical College and Teaching Hospital, Sinamangal, Kathmandu, Nepal; 3 Department of Oral Medicine ond Radiology, Kathmandu Medical College and Teaching Hospital, Sinamangal, Kathmandu, Nepal

**Keywords:** *cusp*, *groove*, *mandibular*, *molar*, *primary tooth*

## Abstract

**Introduction:**

Primary mandibular molars have diverse morphological variations. This trait of human dentition is very important for restoring the anatomy of the tooth as well as for the anthropological study to characterize the ethnicity. Anatomical landmarks like the cusp, groove and ridges vary even among same species. The study was aimed to assess the number of cusps and occlusal grooves in primary mandibular molars of children.

**Methods:**

An observational cross-section study was conducted among pediatric patients in the Outpatient Dental Department of a tertiary care center from October 2023 to March 2024. Fully erupted primary mandibular molars in children aged 3-6 years were included. Intraoral examination of the patient were performed using Gregory WK criteria. A convenience sampling method was used. The point estimate was calculated at a 95% Confidence Interval.

**Results:**

Among 303 patients, the study showed 118 (38.94%) left and 106 (34.98%) right primary mandibular first molar had four cusps and a ‘Y’ groove pattern. Among the 303 subjects, male 58 (34.12%) were having a 4Y pattern followed by 46 (27.06%) 4X pattern, while in 48 (36.09%) 4Y pattern followed by 45 (33.83%) 4X pattern was seen.

**Conclusions:**

The result in present study showed Y groove pattern in occlusal surface of primary mandibular molars with four cusps that can be related with conservative pattern of deciduous molar in context of Nepali population.

## INTRODUCTION

Differences in teeth formation and expression are evident among various populations.^1^ Factors such as culture, environment, and race play a role in shaping the size and shape of teeth.^2^ Variations in the chewing surfaces of lower molars offers valuable insights into population diversity that can aid forensic and anthropological investigations.^3-5^

Researchers have examined 28 distinct dental characteristics in Mongoloid teeth in the first molar of mandible.^6^ Dental anatomy displays diversity due to its significant genetic influence, aiding in evaluation of evolutionary transformations within a population. The occlusal anatomy of primary molars plays a crucial part in both dental anatomy and clinical dentistry.^7^ Difference of tooth morphology should be known to dental practitioners and specialists as it can affect various dental treatments.^8-9^ Hence present study aimed to assess the distribution of number of cusps and occlusal grooves in primary mandibular molars of children visiting dental outpatient department.

## METHODS

This observational cross-section study was conducted among the patients visiting the Outpatient Department of Pedodontics and Preventive Dentistry of Kathmandu Medical College and Teaching Hospital, Duwakot, Bhaktapur, Nepal. Data collection was done from October 2023 to March 2024. Ethical approval was taken from the Institutional Review Committee (Reference number: 18082023/04). Patient's guardians were informed about the procedure and informed consent and assent was obtained. Intraoral examination of the patient was performed in dental chair using diagnostic instruments along with identification of groove pattern following Gregory WK ^10^ criteria. A convenience sampling method was used. Sample size was calculated using the following formula

Sample size (n) = Z^2^p (1-p)/d^2^

= 302.8=303

Where,

n= minimum required sample size
Z = 1.96 at 95% (Confidence interval (CI)
p = 0.73 taken as prevalence of "Y" groove pattern from published article^11^
e = 0.05 (5% margin of error).

Hence, the minimum sample size calculated was 303. Patient vising department of Pedodontics and preventive dentistry with age ranging from 3-6 years having fully erupted primary mandibular second molars were included in the study. Whereas, teeth with developmental anomaly, exposed to trauma, or carious were excluded from the study. Intraoral examination revealed the number of cusp and evaluation of occlusal pattern followed the Gregory WK criteria. Determination of the groove pattern "Y," "+" or "X," if contact of metaconid (mesioligual cusp) with the hypoconid (distobuccal cusp) have been checked. If contact occurs the pattern resembles a "Y" form and if no contact occurs then pattern resembles "+" form and if entoconoid (distoligual cusp) contacts with protoconoid (mesiobuccal cusp) pattern resembles "X" form.^10,11^

Data were entered using Microsoft Excel 2007 and analyzed using IBM SPSS Statistics version 21 (IBM Corp., Armonk, N.Y., USA). The point estimate was calculated at a 95% CI.

## RESULTS

A total of 303 patients were involved in the study. Among them, 170 (56.11%) were male and 133 (43.89%) were female. The present study showed that the 118 (38.94%) of primary mandibular first molars had four cusps, and a groove pattern ‘Y’ (irrespective of side; i.e., 4Y) (Table 1).

**Table 1:** Distribution of number of cusp and groove pattern in left primary mandibular first molar (n=303).

No. of cusp and groove pattern Left primary mandibular first molar	Frequency(%)
4X	79 (26.07)
4Y	118 (38.94)
5X	39 (12.87)
5Y	67 (22.11)
Total	303(100)

When right side of the mandibular first molar were evaluated for the number of cusp and occlusal pattern, 108(35%) showed the similar result i.e: 4Y pattern. (Table 2).

**Table 2:** Distribution of number of cusp and groove pattern in right primary mandibular first molar (n=303).

No. of cusp and groove pattern Right primary mandibular first molar	Frequency (Percentage)
4X	91 (31.03)
4Y	106 (34.98)
5X	42 (13.86)
5Y	62 (20.46)
6Y	2 (0.66)
Total	303 (100)

Crosstabulation was conducted to evaluate the number of cusps and groove pattern among genders. Results showed males had 58 (34.12%) with 4Y pattern followed by 46 (27.06%) with 4X pattern. In females, 48 (36.09%) had 4Y pattern followed by 45 (33.83%) with 4X pattern (Table 3).

**Table 3:** Number of cusp and groove pattern in male and female in mandibular first molars (n=303).

		Frequency(%) Right mandibular molar	Frequency (%) Left mandibular molar
Male	4X	46 (27.06)	44 (25.88)
	4Y	58 (34.12)	67 (39.41)
	5X	26 (15.29)	20 (11.76)
	5Y	38 (22.35)	39 (22.94)
	6Y	2 (1.18)	-
	Total	170 (100)	170 (100)
Female	4X	45 (33.83)	35 (26.32)
	4Y	48 (36.09)	51 (38.35)
	5X	16 (12.03)	19 (14.29)
	5Y	24 (18.05)	28 (21.05)
	Total	133 (100)	133 (100)

## DISCUSSION

Human teeth are important structures. Apart from their obvious role in mastication, they play a vital role in digestion, speech and esthetics. They are also important in genetic research, forensic and anthropological investigations.^8,11^

The occlusal surface of human teeth harbors many anatomical elevations and depressions which have been extensively used in dento-anthropological based identification purposes. These include the number, location and size of cusp and occlusal groove patterns.^12,13^ Cusps and fissures present in the posterior teeth of both maxillary and mandibular arch are essential anatomical structure playing crucial oral functions. Cusps are rounded or pointed elevations that are basically playing role on breaking of food into fine fragments. They are also important for crushing, grinding and tearing of foods. In the oral cavity fissures are basically separating the cusps from each other. They also play a role on guiding food during mastication.^1,3,10^ However, it should also be noted these fissures if present as deep and narrow groove becomes major source of dental caries as food become easily trapped in these narrow grooves, making these areas difficulr to brush.^10^

The formation of teeth is a gradual process that happens over time due to interactions at the molecular and cellular levels. This process involves important developmental milestones that can be impacted by genetic, epigenetic, and environmental elements.^12^ The arrangement of grooves and fissures are unrelated to the count of cusps and is regarded as polygenic, controlled by various alleles at multiple genetic locations.^12^

Numerous studies have shown variations in the morphological pattern in both primary and permanent dentitions among different population. This is due to the influence of the variety of factors including cultural, environmental and racial.^4,6,7^ The structure, dimensions, and quantity of human teeth are undergoing changes. Dahlberg noted that these changes are occurring at different speeds across different racial groups and geographical regions.^7^

Variations in the size, number of cusps, and groove pattern have been observed in the mandibular molars of different populations.^7,8^ There is basic arrangement of cusps and grooves on occlusal surface of primary second molar. In past, various studies have been done to relate the prevalence of dental anatomy with different factors.^7,8,9,11^

Research on dental structure often relies on Gregory and Hellman's classification system. These studies utilized diverse techniques including extracted teeth, intraoral inspections, and dental study models. Hellman classified mandibular molars based on the occlusal pattern and the number of cusps. According to him, the basic pattern is "5y," with five cusps and a "y-"shaped occlusal configuration.^10^ Diverse occlusal character is key feature of molar. Basic arrangement of number of cusp and groove plays a pivotal role for their unique anatomy and clinical presentation. According to the anthropologist "y" groove pattern in molars are conservative whereas "+" pattern is more evolutionary.^13^

In the present study, 170 (56.11%) were male, and 133 (43.89%) were female. When groove patterns were analyzed, irrespective of the sides, the Y pattern was found to be the most common groove pattern. In the left mandibular first molar, 185 (61.05%) teeth had a Y groove pattern, and similarly, 170 (55.77%)of the right first molar had a Y groove. In line with the present study, several other studies have also shown the predominance of the Y groove pattern.

Studies conducted at different places showed Y pattern as predominant groove configuration were 73.34%^11^, 65%^14^, 67%15, 50.36%^16^. The fissure pattern, which develops independently of the number of cusps, is believed to be influenced by multiple genes, involving combinations of alleles at different loci.^17^

In the study, 197 (65.02%) cases showed the 4-cusp form as the most common configuration on both sides, while the 5-cusp pattern was observed in 106 (34.98%) left and 104 (34.32%) right mandibular molars. In contrast with the present study, Ahsana et al. in their study reported predominance of 5 cusps patterns in 96.4% of the population.^15^ Similarly prevalence status of 5 cusps pattern was reported by Felemban et al.^8^ The difference could be due to the selection of tooth type in conducted studies^8,15^ as few of the studies have taken into account of permanent molars^8^ in the other studies while the present study assessed the primary molars. A study done by Hung et al.^18^ on 144 Taiwanese children showed that 93% of primary mandibular first molars had four cusps, 5% had five cusps and 2 % had three cusps while in the study it was lesser than the Taiwanese population. This could be due to genetic variations in these two countries. Both male and female had 4Y as the most predominant feature indicating no gender differences in the study.

In this study, direct intraoral examination of the study subjects was preferred due to its advantages of accurate recording, proper identification of teeth, easy follow-up, less time consuming, minimum armamentarium requirements similar to study.^15^ Studies have also employed analysis of study cast for study of occlusal surface of teeth. Others have also used combination of both methods.^13^

The ultimate shape of a tooth reflects both its genetic makeup and the effects of the environment over time. Primary mandibular first molar has lot of variations in its crown and root morphology. To perform an efficient restoration and pulpal procedures, knowledge of anatomical characteristics and its possible variations is essential.

The present study also has limitations. This is a single centre study so the findings does not generalize all the population of Nepal. The study was conducted with direct intraoral examination method only. However, in the area of forensic odontology and dental anatomy this study has provided a baseline data which is the strength of the present study. There is lack of data on prevalence of dental metrics and traits among Nepalese population, hence there is also need of additional studies.

## CONCLUSIONS

The present study showed Y groove pattern in occlusal surface of deciduous mandibular molars with four cusps that can be related with conservative pattern of primary molar in context of Nepali population. This finding will emphasize on the primitive pattern of evolution of mandibular molar and would help dentists on restorations and forensic purposes.
